# Supplementary material for: Scinderin Is a Novel Oncogene for Its Correlates with Poor Prognosis, Immune Infiltrates and Matrix Metalloproteinase-2/9 (MMP2/9) in Glioma
Source: Brain Sci. 2022 Oct 20;12(10):1415. doi: 10.3390/brainsci12101415 (PMC9599173; doi:10.3390/brainsci12101415)
Supplement: Supplementary file 1 [file brainsci-12-01415-s001.zip › brainsci-1963512-supplementary.pdf]

Table S1. The top 200 SCIN-related genes in LGG and GBM.

Gene Symbol	Gene ID	PCC
NCKAP1L	ENSG00000123338.12	0.88
FYB	ENSG00000082074.15	0.86
VAV1	ENSG00000141968.7	0.86
SASH3	ENSG00000122122.9	0.85
PIK3AP1	ENSG00000155629.14	0.85
SYK	ENSG00000165025.14	0.84
DAPP1	ENSG00000070190.12	0.84
LILRB1	ENSG00000104972.14	0.84
DOCK2	ENSG00000134516.15	0.83
SLC2A5	ENSG00000142583.17	0.83
HCK	ENSG00000101336.12	0.83
PLCG2	ENSG00000197943.9	0.83
APBB1IP	ENSG00000077420.15	0.83
KCNK13	ENSG00000152315.4	0.83
ARHGAP30	ENSG00000186517.13	0.83
LPAR5	ENSG00000184574.9	0.82
SNX20	ENSG00000167208.14	0.82
WAS	ENSG00000015285.10	0.81
CD4	ENSG00000010610.9	0.81
FCGR3A	ENSG00000203747.9	0.81
GPR160	ENSG00000173890.16	0.81
MYO1F	ENSG00000142347.16	0.81
SAMSN1	ENSG00000155307.17	0.8
IL16	ENSG00000172349.16	0.8
RP11-750H9.5	ENSG00000255197.5	0.8
HAVCR2	ENSG00000135077.8	0.8
LILRB4	ENSG00000186818.12	0.8
PTPRC	ENSG00000081237.18	0.8
LYN	ENSG00000254087.7	0.8
RCSD1	ENSG00000198771.10	0.8
ITGB2	ENSG00000160255.16	0.8
ADGRE1	ENSG00000174837.14	0.79
GPSM3	ENSG00000213654.9	0.79
LILRA1	ENSG00000104974.10	0.79
CYTH4	ENSG00000100055.20	0.79
CYBB	ENSG00000165168.7	0.79
CD53	ENSG00000143119.12	0.79
SPI1	ENSG00000066336.11	0.79
ADAP2	ENSG00000184060.10	0.79
AIF1	ENSG00000204472.12	0.79
TMEM106A	ENSG00000184988.8	0.78
ABI3	ENSG00000108798.8	0.78
C1QC	ENSG00000159189.11	0.78
RPS6KA1	ENSG00000117676.13	0.78
HCLS1	ENSG00000180353.10	0.78
CEACAM21	ENSG00000007129.17	0.78
LAPTM5	ENSG00000162511.7	0.78
LINC01146	ENSG00000258867.5	0.78

HLA-DOA	ENSG00000204252.12	0.77
CORO1A	ENSG00000102879.15	0.77
LAT2	ENSG00000086730.16	0.77
KCNQ1	ENSG00000053918.15	0.77
JAK3	ENSG00000105639.18	0.77
C1QB	ENSG00000173369.15	0.77
CTD-3128G10.7	ENSG00000276980.1	0.77
DOCK8	ENSG00000107099.15	0.77
PIK3CG	ENSG00000105851.10	0.77
PTAFR	ENSG00000169403.11	0.77
DENND3	ENSG00000105339.10	0.77
LAIR1	ENSG00000167613.15	0.77
CSF1R	ENSG00000182578.13	0.77
TLR1	ENSG00000174125.7	0.77
TLR7	ENSG00000196664.4	0.76
BTK	ENSG00000010671.15	0.76
POU2F2	ENSG00000028277.20	0.76
BIN2	ENSG00000110934.10	0.76
RGS18	ENSG00000150681.9	0.76
SIGLEC9	ENSG00000129450.8	0.76
FCGR1C	ENSG00000265531.3	0.76
DEF6	ENSG00000023892.10	0.76
NLRC4	ENSG00000091106.18	0.76
ST6GAL1	ENSG00000073849.14	0.75
DOK3	ENSG00000146094.13	0.75
TLR6	ENSG00000174130.12	0.75
IL12RB1	ENSG00000096996.15	0.75
TBXAS1	ENSG00000059377.15	0.75
TM6SF1	ENSG00000136404.15	0.75
IKZF1	ENSG00000185811.16	0.75
GIMAP4	ENSG00000133574.9	0.75
SLCO2B1	ENSG00000137491.14	0.75
TNFRSF1B	ENSG00000028137.16	0.75
STXBP2	ENSG00000076944.14	0.75
HLA-DMA	ENSG00000204257.14	0.75
SLA	ENSG00000155926.13	0.75
WDFY4	ENSG00000128815.17	0.75
C16orf54	ENSG00000185905.3	0.74
FPR1	ENSG00000171051.8	0.74
RHBDF2	ENSG00000129667.12	0.74
NAGA	ENSG00000198951.11	0.74
ALOX5	ENSG00000012779.10	0.74
CCR1	ENSG00000163823.3	0.74
ARHGDIB	ENSG00000111348.8	0.74
TLR2	ENSG00000137462.6	0.74
TMC8	ENSG00000167895.14	0.74
C3AR1	ENSG00000171860.4	0.74
MNDA	ENSG00000163563.7	0.74
MGAT4A	ENSG00000071073.12	0.74
ITGAL	ENSG00000005844.17	0.74
CCR5	ENSG00000160791.13	0.74

CD68	ENSG00000129226.13	0.74
RP11-153M7.1	ENSG00000248208.1	0.74
LHFPL2	ENSG00000145685.13	0.74
FAM177B	ENSG00000197520.10	0.74
ARL11	ENSG00000152213.3	0.74
RASGRP4	ENSG00000171777.15	0.74
EBI3	ENSG00000105246.5	0.74
TNFAIP8L2	ENSG00000163154.5	0.74
UCP2	ENSG00000175567.8	0.74
PCED1B-AS1	ENSG00000247774.6	0.73
SIRPB2	ENSG00000196209.12	0.73
DENND1C	ENSG00000205744.9	0.73
FCGR1A	ENSG00000150337.13	0.73
PARVG	ENSG00000138964.16	0.73
TLR10	ENSG00000174123.10	0.73
FCGR1B	ENSG00000198019.12	0.73
MILR1	ENSG00000271605.5	0.73
LY86	ENSG00000112799.8	0.73
TLR5	ENSG00000187554.11	0.73
LPCAT2	ENSG00000087253.11	0.73
CD84	ENSG00000066294.14	0.73
PLB1	ENSG00000163803.12	0.73
ADORA3	ENSG00000282608.1	0.73
SIGLEC10	ENSG00000142512.14	0.73
CD86	ENSG00000114013.15	0.73
GNGT2	ENSG00000167083.6	0.72
CD37	ENSG00000104894.11	0.72
CD300C	ENSG00000167850.3	0.72
CD74	ENSG00000019582.14	0.72
GPR65	ENSG00000140030.5	0.72
IL10RA	ENSG00000110324.9	0.72
GPR132	ENSG00000183484.11	0.72
CD300A	ENSG00000167851.13	0.72
HLA-DMB	ENSG00000242574.8	0.72
ARHGAP9	ENSG00000123329.17	0.72
RNASE3	ENSG00000169397.3	0.72
CSF2RB	ENSG00000100368.13	0.72
PLXDC2	ENSG00000120594.16	0.72
RP11-83C7.1	ENSG00000248227.1	0.72
RGS19	ENSG00000171700.13	0.72
FAM78A	ENSG00000126882.12	0.71
LRRC25	ENSG00000175489.9	0.71
CD14	ENSG00000170458.13	0.71
AC011899.9	ENSG00000233038.5	0.71
LGALS9	ENSG00000168961.16	0.71
HLA-DRA	ENSG00000204287.13	0.71
EVI2B	ENSG00000185862.6	0.71
LCP1	ENSG00000136167.13	0.71
CD226	ENSG00000150637.8	0.71
CEBPA	ENSG00000245848.2	0.71
C1QA	ENSG00000173372.16	0.71

RIPK3	ENSG00000129465.15	0.71
ATP8B4	ENSG00000104043.14	0.71
TRAF3IP3	ENSG00000009790.14	0.7
CTC-378H22.1	ENSG00000254887.1	0.7
IRF5	ENSG00000128604.18	0.7
ITGAM	ENSG00000169896.16	0.7
FERMT3	ENSG00000149781.12	0.7
GPR34	ENSG00000171659.13	0.7
CASP5	ENSG00000137757.10	0.7
HLA-DPA1	ENSG00000231389.7	0.7
AC098823.3	ENSG00000226125.1	0.7
CYFIP1	ENSG00000273749.4	0.7
RP11-23J18.1	ENSG00000258352.1	0.7
IL21R	ENSG00000103522.15	0.7
RNASET2	ENSG00000026297.15	0.7
RP11-153M7.3	ENSG00000250771.2	0.7
NFAM1	ENSG00000235568.6	0.69
EMB	ENSG00000170571.11	0.69
NCF4	ENSG00000100365.14	0.69
TMEM119	ENSG00000183160.8	0.69
SCIMP	ENSG00000161929.14	0.69
AOAH	ENSG00000136250.11	0.69
PLA2G15	ENSG00000103066.12	0.69
GBGT1	ENSG00000148288.11	0.69
FCER1G	ENSG00000158869.10	0.68
PIK3R5	ENSG00000141506.13	0.68
CYBA	ENSG00000051523.10	0.68
RASAL3	ENSG00000105122.12	0.68
HLA-DRB6	ENSG00000229391.7	0.68
HPGDS	ENSG00000163106.10	0.68
CARD9	ENSG00000187796.13	0.68
CD33	ENSG00000105383.14	0.68
HHEX	ENSG00000152804.10	0.68
RP11-344B5.2	ENSG00000224307.1	0.68
GIMAP6	ENSG00000133561.15	0.68
FAM105A	ENSG00000145569.5	0.68
BATF	ENSG00000156127.6	0.68
RHOH	ENSG00000168421.12	0.68
CIITA	ENSG00000179583.17	0.68
IRF8	ENSG00000140968.10	0.68
TGFBR1	ENSG00000106799.12	0.68
HLA-DPB1	ENSG00000223865.10	0.68
CLEC17A	ENSG00000187912.11	0.68
BLNK	ENSG00000095585.16	0.68
IL6R	ENSG00000160712.12	0.68
GIMAP1	ENSG00000213203.2	0.67
ZC3H12D	ENSG00000178199.13	0.67
LCP2	ENSG00000043462.11	0.67
SYNGR2	ENSG00000108639.7	0.67
AC006129.2	ENSG00000268027.5	0.67

Notes: PCC, Pearson correlation coefficient. SCIN, Scinderin. LGG, lower grade glioma. GBM, glioblastoma
